# 染色体3p区抑癌基因在非小细胞肺癌中的甲基化状况与临床意义

**DOI:** 10.3779/j.issn.1009-3419.2011.03.09

**Published:** 2011-03-20

**Authors:** 海珠 宋, 俊 易, 有为 张, 锐 王, 龙邦 陈

**Affiliations:** 1 210002 南京，第二军医大学南京临床学院（南京军区南京总医院）肿瘤内科 Department of Medical Oncology, Jinling Hospital, Nangjing Clinical School of the Second Military Medical University, PLA, Nanjing 210002, China; 2 210002 南京，第二军医大学南京临床学院（南京军区南京总医院）心胸外科 Department of Cardiothoracic Surgery, Jinling Hospital, Nangjing Clinical School of the Second Military Medical University, PLA, Nanjing 210002, China

**Keywords:** 肺肿瘤, 染色体, 甲基化, 生物标记, Lung neoplasms, Chromosome, Methylation, Biomarker

## Abstract

**背景与目的:**

DNA甲基化是表观遗传学的一种调控机制，染色体3p区等位基因缺失是肺癌发生中较频繁和早期的事件之一。检测染色体3p区5个典型抑癌基因*DLEC1*、*RASSF1A*、*hMLH1*、*RARβ*和*FHIT*在非小细胞肺癌（non-small cell lung cancer, NSCLC）中的甲基化状况，分析其临床意义。

**方法:**

取78例NSCLC患者术中癌组织及相应正常肺组织标本，采用甲基化特异性聚合酶链反应（methylation specific PCR, MSP）检测基因启动子区甲基化状况，RT-PCR和免疫组化检测*DLEC1*基因表达。

**结果:**

78例NSCLC组织中，*DLEC1*、*RASSF1A*、*RARβ*和*hMLH1*甲基化频率分别为41.03%、39.74%、30.77%和16.67%，与正常组织相比差异均具有统计学意义。*FHIT*基因在癌组织和正常组织均无甲基化。*DLEC1*甲基化与患者临床分期（*P*=0.011）和淋巴结转移相关（*P*=0.019），而*RASSF1A*、*RARβ*、*hMLH1*基因甲基化以及平均甲基化指数与临床病理特征无关联。56.41%（44/78）的NSCLC组织中发现*DLEC1*基因表达下调或缺失，且与启动子甲基化有关。

**结论:**

3p区抑癌基因甲基化是NSCLC发生中的重要分子事件，可能作为NSCLC早期诊断的潜在生物标记，新型抑癌基因*DLEC1*失活与启动子高甲基化有关。

## Abstract (en)

**Background and objective:**

DNA methylation is one of the mechanisms of epigenetics. Allelic loss located on chromosome 3p happen frequently and early in non-small cell lung cancer (NSCLC). The aim of this study is to detect the promoter methylation status of tumor suppressor genes (TSGs) located on chromosome 3p in NSCLC and to evaluate its correlation with clinicopathological features.

**Methods:**

A total of 78 paired NSCLC specimens and their adjacent normal tissues were collected in the study. Promoter methylation status was determined by methylation-specific polymerase chain reaction (MSP). *DLEC1* gene expression was determined by RT-PCR and immunohistochemistry.

**Results:**

Aberrant methylation frequency of *DLEC1*, *RASSF1A*, *hMLH1*, *RARβ* and *FHIT* genes detected in 78 NSCLC tissues were 41.03%, 39.74%, 30.77% and 16.67%, respectively, which were all significantly higher than that in adjacent normal tissues. However, *FHIT* gene was not detected methylation in both cancerous and non-cancerous tissues. *DLEC1* hypermethylation was associated with advanced stage (*P*=0.011) and lymph metastasis (*P*=0.019), while *RASSF1A*, *RARβ*, *hMLH1* and mean methylation index (MI) were not correlated with any clinicopathological parameters. Moreover, *DLEC1* gene downregulation was detected in 56.41% (44/78) NSCLC tissues and correlated with promoter hypermethylation.

**Conclusion:**

Frequent hypermethylation of TSGs located on chromosome 3p was a common event contributing to NSCLC pathogenesis and *DLEC1* methylation was closely correlated with loss of expression.

DNA甲基化是表观遗传学的一种调控机制，抑癌基因启动子区域CpG岛的异常高甲基化，使染色质螺旋程度增加，基因转录受到抑制，与肿瘤发生密切相关^[1]^。肺癌，以非小细胞肺癌（non-small cell lung cancer, NSCLC）为主，是世界范围内癌症致死的首要原因。研究NSCLC特异的DNA甲基化将为揭示其发病机制提供线索并可能作为潜在的生物标记。

染色体3p区等位基因缺失是肺癌发生中较频繁和早期的事件之一，其中3p12-13、3p14.2、3p21.1-21.2、3p21.3和3p24-26等被证实是缺失的热点区域，提示在这些区域存在多个抑癌基因^[2, 3]^。本实验以改良的甲基化特异性聚合酶链反应（methylation specific PCR, MSP）检测78例NSCLC组织中3p区抑癌基因*DLEC1*（deleted in lung and esophageal cancer 1）、*RASSF1A*（Ras associated domain family member 1）、*hMLH1*（mutL homolog 1）、*RARβ*（retinoic acid receptor β）和*FHIT*（fragile histidine triad gene）的甲基化状况，并分析与临床病理特征的关系。

## 1 材料与方法

### 1.1 标本

78例NSCLC组织标本（癌组织及癌旁>5 cm处或切缘处正常组织）源自2007年11月-2008年7月于南京军区南京总医院心胸外科行手术治疗者。所有患者均经病理检查确诊，术前未行放射治疗或化学治疗，其中男58例，女20例，年龄35岁-80岁，中位年龄59岁。根据国际抗癌联盟（Universal Integrated Circuit Card, UICC）第7版指南进行分期，其中Ⅰ期25例，Ⅱ期33例，Ⅲ期19例，Ⅳ期1例（脑转移）。

### 1.2 DNA提取

采用QIAamp DNA Mini Kit试剂盒（德国Qiagen公司）提取组织DNA。操作严格依照说明书进行。

### 1.3 亚硫酸氢盐修饰

采用EZ DNA Methylation-GOLD Kit（D5006）试剂盒（美国Zymo Research）。组织DNA经分光光度计测定浓度后取1 μg进行修饰。最后以10 μL M-Elution Buffer洗脱DNA，-80 ℃保存。经此步后，DNA序列中未甲基化的胞嘧啶（C）转变为尿嘧啶（U）。

### 1.4 采用MSP法检测基因启动子区甲基化状况

各引物序列及反应条件见表 1。PCR反应体系25 μL，其中10× PCR buffer 2.5 μL（含Mg^2+^，终浓度为1.5 mmol/L），dNTP mixture 2.5 μL（终浓度250 μmol/L），上下游引物各2 μL（30 pmol），修饰后的DNA模板5 μL，灭菌去离子水10.85 μL，Taq酶0.15 μL（日本Takara公司）。正常人外周血淋巴细胞DNA作为非甲基化阳性对照，过量CpG（SssI）甲基化酶（美国New England Biolabs公司）修饰的淋巴细胞DNA作为甲基化阳性对照，ddH_2_O代替DNA作为阴性对照。

**1:** 引物序列 List of primer sequences

Primer	Sense (5’-3’)	Antisense (5’-3’)	Product	Temperature (Cycle)
*FHIT* (M)	GAAGGTAGG GGCGGGGAGGTAAGTT	CGTAAACGACGCCGACCCCACTA	116 bp	68 ℃ (40)
*FHIT* (U)	GAAGGTAGG GGTGGGGAGGTAAGTT	CATAAACAACACCAACCCCACTA	116 bp	63 ℃ (40)
*RASSF1A* (M)	GGGTTTTGCGAGAGCGCG	GCTAACAAACGCGAACCG	169 bp	64 ℃ (40)
*RASSF1A* (U)	GGTTTTGTGAGAGTGTGTTTAG	CACTAACAAACACAAACCAAAC	169 bp	59 ℃ (40)
*hMLH1* (M)	TTAATAGGAAGAGCGGATAGC	CTATAAATTACTAAATCTCTTCG	106 bp	55 ℃ (40)
*hMLH1* (U)	TTAATAGGAAGAGTG GATAGTG	TCTATAAATTACTAAATCTCTTCA	107 bp	55 ℃ (40)
*DLEC1* (M)	GATTA AGCGATGACGGGATTC	ACC CGACTAATAACGAAATTAACG	193 bp	60 ℃ (40)
*DLEC1* (U)	TGATTATAGTGATGATGG GATTTGA	CCCAAC TAATAACAAAATTAACACC	193 bp	60 ℃ (40)
*RARβ* (M)	TCGAGAACGCGAGCGATTCG	GACCAATCCAACCGAAACGA	146 bp	62 ℃ (40)
*RARβ* (U)	TTGAGAATGTGAGTGATTTGA	AACCAATCCAACCAAAACAA	146 bp	62 ℃ (40)
*DLEC1* (R)	TTCCTCCCTCGCCTACTC	AAACTCATCCAGCCGCTG	309 bp	55 ℃ (30)
*GAPDH* (R)	CAATGACCCCTTCATTGACC	TGGAAGATGGTGATGGGATT	135 bp	55 ℃ (30)
M: Methylated; U: Unmethylated; R: RT-PCR.				

### 1.5 采用RT-PCR检测*DLEC1*基因的表达

采用Trizole一步法进行组织RNA的抽提，3 μL RNA样品用1%琼脂糖凝胶电泳检测RNA的完整性。RNA定量后取2 μg进行逆转录反应（first strand cDNA kit, Takara），之后以25 μL反应体系进行体外PCR扩增。*DLEC1*及*DAPDH*引物见表 1。PCR产物于2%琼脂糖凝胶中电泳，在图像扫描仪下观察，用SmartView生物电泳图像分析系统处理，并用同一样品的*GAPDH*扩增产物作为内参照进行校正，得出目的条带与内参照条带的吸光度比值。

### 1.6 免疫组织化学法检测*DLEC1*基因的表达

石蜡包埋切片经脱蜡、水化、抗原修复后，EnVision二步法检测DLEC1蛋白表达，兔抗人DLEC1单抗（Sigma公司，1:200稀释）4 ℃孵育过夜，PBS冲洗，二抗（Dako, Ely, UK）室温孵育30 min，DAB显色，苏木素复染。实验同时以PBS代替一抗作空白对照。DLEC1蛋白表达定位于细胞浆，为黄-棕黄色颗粒，采用半定量积分法判定结果。于高倍镜下随机选取5个视野（每个视野观察细胞数不少于200个），具体标准如下：（1）阳性细胞数0-5%为0分，6%-25%为1分，26%-50%为2分，>50%为3分；（2）阳性强度无色为0分，淡黄色为1分，棕黄色为2分，棕褐色为3分。将（1）（2）两者积分相加，癌组织总分低于相应正常组织者为低表达。

### 1.7 统计学方法

采用SPSS 12.0软件进行统计分析，结果以Mean±SD或百分比表示。*DLEC1*表达差异采用配对*t*检验；率的比较采取*χ*^2^检验或*Fisher*确切概率法；甲基化指数（methylation index, MI)，定义为每个样本发生甲基化的位点与检测的总位点的比值，组间差异比较行方差分析（ANOVA）。*P* < 0.05为差异有统计学意义。

## 2 结果

### 2.1 NSCLC组织甲基化状况

*DLEC1*、*RASSF1A*、*RARβ*和*hMLH1*在78例NSCLC肿瘤组织中的甲基化频率分别为41.03%、39.74%、30.77%和16.67%，而在相应正常组织中为3.85%、7.69%、8.97%和5.13%，差异均具有统计学意义（表 2）。联合检测这4个基因甲基化在肿瘤组织中的阳性率达到69.23%，正常组织中为16.67%（*P* < 0.001）。*FHIT*基因在所检测的40例样本中，无论癌组织还是正常组织均无甲基化。典型MSP结果见图 1。

**2:** 非小细胞肺癌组织和相应正常组织甲基化差异(*n* =78) Methylation profiles in NSCLC tissues and matched normal tissues (*n* =78)

Gene	Methylation frequency [*n* (%)]		95%CI	P^*^
	Tumor tissues	Normal tissues		
*DLEC1*	32 (41.03)	3 (3.85)	2.207-126.864	< 0.001
*RASSF1A*	31 (39.74)	6 (7.69)	3.066-20.431	< 0.001
*RARβ*	24 (30.77)	7 (8.97)	1.809-11.236	0.001
*hMLH1*	13 (16.67)	4 (5.13)	1.149-11.911	0.037
*FHITa*	0 (0)	0 (0)	—	—
*DLEC1*+*RASSF1A*+*RARβ*+*hMLH1*	54 (69.23)	13 (16.67)	5.233-24.185	< 0.001
^*^Chi-square test or *Fisher’s* exact test; ^a^*n* =40.				

**1:**
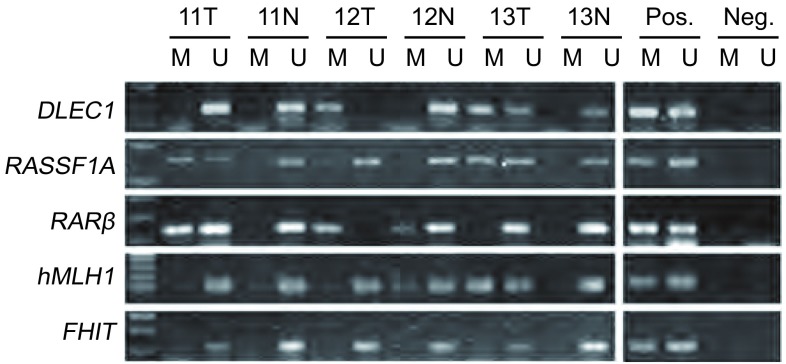
MSP法检测肿瘤组织和正常组织甲基化的结果（病例11-13）。T：肿瘤组织；N：正常组织；M：甲基化；U：未甲基化；Pos：阳性对照；Neg：阴性对照 Representative MSP profiles in matched NSCLC and adjacent normal lung tissues (cases 11-13). T: tumor tissues; N: normal tissues; M: methylation; U: unmethylation; Pos: positive control; Neg: negtive control

### 2.2 DNA甲基化与临床病理特征的相关性

*DLEC1*基因甲基化与NSCLC临床分期（Ⅰ/Ⅱ *vs* Ⅲ/Ⅳ: 19/58 *vs* 13/20; *P*=0.011）和淋巴结转移（N0 *vs* N1/N2/N3: 13/54 *vs* 19/34;*P*=0.019）有关，在不同年龄、性别、分化程度、肿瘤大小和是否吸烟的患者之间，*DLEC1*启动子异常甲基化检出率无差异。而*RASSF1A*、*RARβ*、*hMLH1*基因甲基化以及平均甲基化指数与临床病理特征无关联（表 3）。

**3:** 3p区抑癌基因甲基化与非小细胞肺癌患者临床病理特征的关系 Association between the DNA methylation in NSCLC specimens and clinicopathological features

Characteristic	*n*	Methylation frequency [*n* (%)]				Mean MI
		*DLEC1*	*RASSF1A*	*RARβ*	*hMLH1*	
Gender						
Male	58	22 (37.93)	24 (41.38)	16 (27.59)	9 (15.52)	0.306±0.116
Female	20	10 (50.00)	7 (35.00)	8 (40.00)	4 (20.00)	0.363±0.125
Age (year)						
< 60	22	8 (36.36)	6 (27.27)	5 (22.76)	3 (13.64)	0.341±0.095
≥60	56	24 (42.86)	25 (44.64)	19 (33.93)	10 (17.86)	0.348±0.122
Histological type						
Adenocarcinoma	30	12 (40.0)	14 (46.67)	8 (26.67)	4 (13.33)	0.317±0.148
Squamous cell carcinoma	36	16 (44.44)	12 (33.33)	14 (38.89)	8 (22.22)	0.347±0.095
Others	12	4 (33.33)	5 (41.67)	2 (16.67)	1 (8.33)	0.250±0.152
Tumor size						
≤3 cm	25	9 (36.0)	11 (44.0)	5 (20.0)	3 (12.0)	0.280±0.146
>3 cm	53	23 (43.40)	20 (37.74)	19 (35.85)	10 (18.87)	0.349±0.106
Cellular differentiation						
Well	13	4 (30.78)	6 (46.16)	3 (23.08)	2 (15.38)	0.288±0.131
Moderate	46	17 (36.95)	18 (39.13)	15 (32.61)	7 (15.21)	0.310±0.108
Poor	19	11 (57.89)	8 (42.11)	6 (31.58)	4 (21.05)	0.382±0.157
Stage						
Ⅰ/Ⅱ	58	19 (32.76)	22 (37.93)	18 (31.03)	8 (13.79)	0.289±0.105
Ⅲ/Ⅳ	20	13 (65.00)^*^	9 (45.00)	6 (30.00)	5 (25.00)	0.412±0.179
Lymph metastasis						
N0	44	13 (29.55)	17 (38.64)	12 (27.27)	7 (15.90)	0.278±0.093
N1/N2 /N3	34	19 (55.88)^*^	14 (41.18)	12 (35.29)	6 (17.64)	0.375±0.158
Smoking habit						
Smoker	49	18 (36.73)	20 (40.82)	17 (34.69)	7 (14.28)	0.316±0.118
Never	29	14 (48.28)	11 (37.93)	7 (24.14)	6 (20.69)	0.328±0.127
^*^P < 0.05						

### 2.3 *DLEC1*基因表达状况

新型抑癌基因*DLEC1*在78例NSCLC组织中的表达通过RT-PCR（图 2）和免疫组化（图 3）鉴定。与相应正常组织相比，44例（56.41%）肿瘤组织的*DLEC1*基因在mRNA和蛋白水平均表达下调或缺失；并且，在32例甲基化的肿瘤组织中，28例表现出*DLEC1*下调或缺失，表明*DLEC1*基因失活与启动子高甲基化密切相关（表 4，*P* < 0.001）。

**2:**
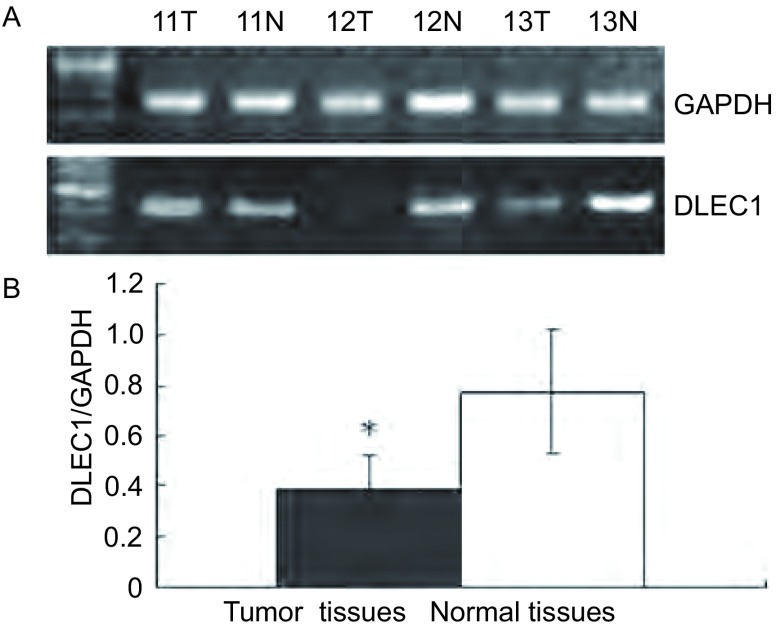
*DLEC1*在非小细胞肺癌中的mRNA表达。A：典型PCR电泳结果（病例11-13）；B：肿瘤组织中DLEC1平均吸光度比值低于相应正常组织。*P < 0.001 mRNA expression levels of *DLEC1* in NSCLC tissues determined by RT-PCR. A: Typical gel electrophoresis results in three matched pairs (cases 11-13) of tumor (T) and their adjacent normal lung tissues (N); B: Histogram of the relative mRNA expression level of *DLEC1* in NSCLC and their adjacent normal tissues. *P < 0.001

**3:**
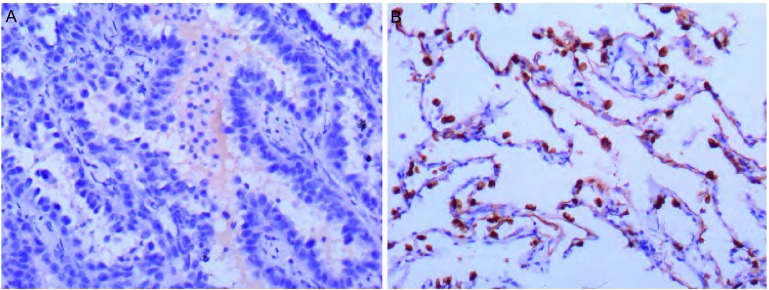
DLEC1在非小细胞肺癌中的蛋白表达（病例12，EnVision法，×200）。A：腺癌组织呈阴性表达；B：正常肺组织阳性表达 Protein expression levels of DLEC1 in NSCLC determined by immunohistochemical staining (case 12, EnVision, ×100). DLEC1 protein was silenced in adenocarcinoma tissues (A), while widely expressed in adjacent normal lung tissues (B)

**4:** *DLEC1*基因在非小细胞肺癌中的表达与启动子甲基化的关系 *DLEC1* downregulation in NSCLC tissues was associated with promoter methylation

*DLEC1* Methylation	*DLEC1* expression		*P*
	Downregulation/Silence	Upregulation/-	
Methylation	28	4	< 0.001
Unmethylation	16	30	

## 3 讨论

NSCLC的发生是多步骤的过程，涉及癌基因的激活和抑癌基因的失活。除了基因水平上的突变、缺失等，表观遗传学的改变，特别是DNA的5’CpG岛区域异常甲基化是导致抑癌基因失活的主要原因之一。由于表观遗传学的改变常发生于遗传学改变之前，因此在NSCLC发生的早期就可能检测到抑癌基因异常甲基化^[4]^，这对于肿瘤的早期诊断具有重要意义。

3号染色体短臂（3p）被认为是多个抑癌基因“停泊”的港口，本研究在78例NSCLC组织中检测5个典型的3p区抑癌基因甲基化情况，包括位于3p21.3-22的*DLEC1*，3p21.3的*RASSF1A*、*hMLH1*，3p24的*RARβ*和3p14.2的*FHIT*。结果发现，*DLEC1*、*RASSF1A*、*RARβ*和*hMLH1*在癌组织的甲基化频率依次为41.03%、39.74%、30.77%和16.67%，均显著高于相应正常组织。并且，69.23%的肿瘤组织至少发生这四者之中一个位点的甲基化，而正常组织仅为16.67%。其中，*DLEC1*是新近在肺癌、食管癌和肾癌中鉴定的候选抑癌基因，转染*DCEC1*基因到肿瘤细胞株中明显抑制细胞克隆形成及体内致瘤性^[5, 6]^，但其抑癌作用的机制尚不明确；*RASSF1A*是Ras激活信号传导通路的负向调节因子，可阻断Ras生长效应信号由胞外传向胞内^[7]^；*RARβ*是维甲酸（retinoic acid, RA）受体，参与抑制维甲酸介导的细胞增殖和分化^[8]^；*hMLH1*属于错配修复基因，对维护基因组的稳定性起着重要的作用^[9]^。它们在NSCLC中的异常甲基化均有报道，本实验进一步证实其在中国人群中的普遍性，可能是NSCLC发病的重要机制。

值得注意的是，*FHIT*基因参与DNA修复及细胞凋亡抑制，在NSCLC中的表达下调及启动子高甲基化亦有诸多报道^[8, 10]^，但在本实验检测的40例样本中，*FHIT*在癌组织和正常组织均无异常甲基化。这可能与样本异质性有关，除甲基化之外，尚有其它因素如微卫星不稳定（microsatellite instability, MSI）和杂合性缺失（loss of heterozygosity, LOH）等导致*FHIT*基因失活^[11]^。

进一步的分析表明，*DLEC1*基因甲基化与NSCLC临床分期和淋巴结转移相关，提示*DLEC1*启动子甲基化还参与肿瘤演进，可能作为潜在的预后指标。但*RASSF1A*、*RARβ*、*hMLH1*基因甲基化以及包括*DLEC1*在内的四者平均MI与NSCLC临床病理特征无关联，表明3p区抑癌基因甲基化是一个相对独立的危险因素，在总体水平上仍是NSCLC发生中的早期事件，可作为NSCLC早期诊断的潜在标记物。

新型抑癌基因*DLEC1*包含37个外显子，长约59 kb，编码一个含有1, 755个氨基酸的蛋白，这个蛋白与已知的所有蛋白都没有相近的同源序列^[5]^，其在人类正常组织中广泛表达，而在大多数肿瘤组织和细胞系中表达下调。目前的研究^[6, 12-15]^表明启动子高甲基化可能是导致*DLEC1*基因失活的重要原因，如*DLEC1*在大部分肝癌细胞系和70.6%（48/68）的原发肝癌中表现出高甲基化，而在临近正常肝组织中仅为10.3%（7/68），去甲基化药物5-Aza-dC处理可使肝癌细胞系恢复*DLEC1*表达^[6]^；鼻咽癌中，71%（30/42）的原发肿瘤检出*DLEC1*高甲基化，且所有*DLEC1*表达沉默的肿瘤细胞系均为高甲基化，而*DLEC1*充分表达的鼻咽部上皮细胞均为未甲基化^[13]^。在肺癌组织中，Seng等^[16]^也发现，38.9%（93/239）的NSCLC中存在*DLEC1*高甲基化，并与患者临床分期、淋巴结转移和不良预后有关。本研究进一步证实，56.41%（44/78）的NSCLC组织中，*DLEC1*基因在mRNA和蛋白水平均表达下调或缺失，并且，*DLEC1*基因失活与启动子高甲基化密切相关，有助于进一步揭示其在NSCLC发生发展中的作用。

总之，本研究证实3p区抑癌基因甲基化是NSCLC发生中的重要分子事件，新型抑癌基因*DLEC1*基因失活与启动子高甲基化有关。
